# Analyses of the Redistribution of Work following Cardiac Resynchronisation Therapy in a Patient Specific Model

**DOI:** 10.1371/journal.pone.0043504

**Published:** 2012-08-28

**Authors:** Steven Alexander Niederer, Pablo Lamata, Gernot Plank, Phani Chinchapatnam, Matt Ginks, Kawal Rhode, Christopher Aldo Rinaldi, Reza Razavi, Nicolas Peter Smith

**Affiliations:** 1 Imaging Sciences & Biomedical Engineering Division, King’s College London, London, United Kingdom; 2 Computing Laboratory, University of Oxford, Oxford, United Kingdom; 3 Institut für Biophysik, Medizinische Universität Graz, Graz, Austria; 4 Department of Cardiology, St Thomas’ Hospital, London, United Kingdom; University Hospital of Würzburg, Germany

## Abstract

Regulation of regional work is essential for efficient cardiac function. In patients with heart failure and electrical dysfunction such as left branch bundle block regional work is often depressed in the septum. Following cardiac resynchronisation therapy (CRT) this heterogeneous distribution of work can be rebalanced by altering the pattern of electrical activation. To investigate the changes in regional work in these patients and the mechanisms underpinning the improved function following CRT we have developed a personalised computational model. Simulations of electromechanical cardiac function in the model estimate the regional stress, strain and work pre- and post-CRT. These simulations predict that the increase in observed work performed by the septum following CRT is not due to an increase in the volume of myocardial tissue recruited during contraction but rather that the volume of recruited myocardium remains the same and the average peak work rate per unit volume increases. These increases in the peak average rate of work is is attributed to slower and more effective contraction in the septum, as opposed to a change in active tension. Model results predict that this improved septal work rate following CRT is a result of resistance to septal contraction provided by the LV free wall. This resistance results in septal shortening over a longer period which, in turn, allows the septum to contract while generating higher levels of active tension to produce a higher work rate.

## Introduction

Cardiac resynchronization therapy (CRT) is an established treatment for patients with heart failure (HF) and dysnchrnous contraction usually manifested on the surface ECG as left branch bundle block (LBBB). Not only does this treatment improve heart function, quality of life and patient morbidity, it also results in homogenization of the distributions of perfusion, contraction, glucose metabolism and oxidative metabolism across the heart [1], [2], [3], [4]. Because, all four of these distributions are strongly correlated [3], [5], [6], [7], this also suggests CRT should produce a more uniform spatial distribution of energy consumption across the heart.

Previous studies have demonstrated that oxygen consumption, or analogously energy consumption, is indicative of the work performed by a muscle [8], [9]. Thus, the observed changes following CRT are likely to be partially attributable to a redistribution of mechanical work across the heart [1]. These changes in cardiac oxygen uptake occur immediately following CRT [10] as a result of an acute improvement in contractile timing. This suggests that part of the change in the distribution of energy consumption is a result of the post-operative changes in the distribution of mechanical work in the heart.

The mechanical work performed by the heart is the result of the integrated work performed by the constituent myocytes. These cellular level work contributions can be characterized by three different work modes. Firstly, myocytes may not be generating active-tension, corresponding to either scarred/stunned or inactivated myocytes. Secondly, myocytes can generate tension resulting in their contraction. Thirdly, myocytes that are generating tension can be stretched by other contracting, tension-generating myocytes. These three modes will be referred to as non-working, positive work and negative work, respectively [11], [12]. Positive work at the cellular level results in a combination of three outcomes: the reduction of the cavity volumes (pump function), the passive deformation of the myocardium and/or stretching of tension-generating myocytes. Positive work used to deform the passive myocardium will largely be retrieved at another stage of the cardiac cycle as the heart is approximately elastic [13] and so does not affect the efficiency of the heart. Positive work used to stretch tension generating myocytes (which are producing negative work) will be dissipated when the stretched cross bridges unbind from actin. Any negative work performed on a myocyte to stretch bound cross bridges will be lost, resulting in a decrease in myocardial efficiency.

Changes in the spatial distribution of non-working, positive work and negative work myocytes across the heart and the magnitude of the work performed by the myocytes in each of these regions following CRT will thus alter the function of the heart. The spatial and temporal dynamics of work rate is determined by the regional distributions of active-tension and the strain rate (SR). Hence any changes in work distribution following CRT must be considered in the context of the multi-scale regulation of these cell level properties.

There is no single imaging modality that can readily determine the distribution of local active tension or work in the heart. However, by integrating multiple diagnostic data sets into a biophysical computational modeling framework the distribution of both work and active tension can be calculated. In this study we have applied this approach to create a personalized human heart model that allows us to calculate regional work pre and post CRT. We use the model to determine if changes in work with CRT are due to changes in the size of the regions performing positive or negative work or if it is due to changes in the magnitude of the work rate in each region. We then use the model to determine if the changes in regional work rate with CRT are due to changes in the strain rate or active tension. Finally we use the model to differentiate the changes in distinct regions of the LV free wall with pacing.

## Methods

### Clinical Protocol

All data was obtained from a 60 year old female with NYHA Class III HF despite optimal medical treatment. There was significant left ventricular (LV) systolic dysfunction with an LV ejection fraction of 25%. Mitral regurgitation was trivial. The surface ECG demonstrated significant conduction disease with LBBB morphology and QRS duration of 154 ms. Cardiac MRI showed a small area of subendocardial apical septum scar and severe LV dysfunction. During catheterisation the sinus heart rate was 69 bpm.

The study complied with the South East London Ethics Committees, adheres to the declaration of Helsinki and informed consent was obtained from the patient**.** Bilateral femoral venous access was used to place quadripolar catheters (St Jude Medical, USA) to the high right atrium and RV apex to perform RV atrial and ventricular pacing. The coronary sinus was catheterised and a multipolar catheter (Cardima, USA) was passed to a postero-lateral branch of the coronary sinus to perform epicardial LV pacing to replicate standard CRT. An Ensite™ 3000 non-contact mapping array (St Jude Medical, USA) was passed via the femoral artery retrogradely across the aortic valve to the LV cavity. Via the other femoral artery, a decapolar catheter (St Jude Medical, USA) was passed to the LV cavity along with a high fidelity pressure wire (Radi Medical Systems, Sweden) acquiring data at 500 Hz.

A pacing protocol was then performed as follows (100 bpm, AV delay 100 ms where appropriate, VV simultaneous): Atrial, RV and LV. For our patient simulation, LV coronary sinus pacing was used. AV delay of 100 ms resulted in fusion of the paced activation wave with the intrinsic activation and was observed in the Ensite activation maps. The VV delay was simultaneous to best approximate a healthy synchronous activation. Hemodynamic and electrophysiological parameters were assessed at baseline and once steady state pacing had been achieved for a minimum of one minute. The electrograms were acquired at 1200 Hz. The high pass filter was set at 8 Hz. The onset of activation was defined as the first peak negative unipolar electrogram at any point in the LV. The QRS duration was obtained from the surface 12 lead ECG.

The presence of the catheters in the LV (pressure or Ensite) could affect contraction. As the patients LV cavity is large and dilated, the most likely affect is through ectopic beats. To reduce these effects, recordings are made when the catheters are stable within the LV and we exclude any recordings with ectopic activity.

### Model Development

**Figure 1:**
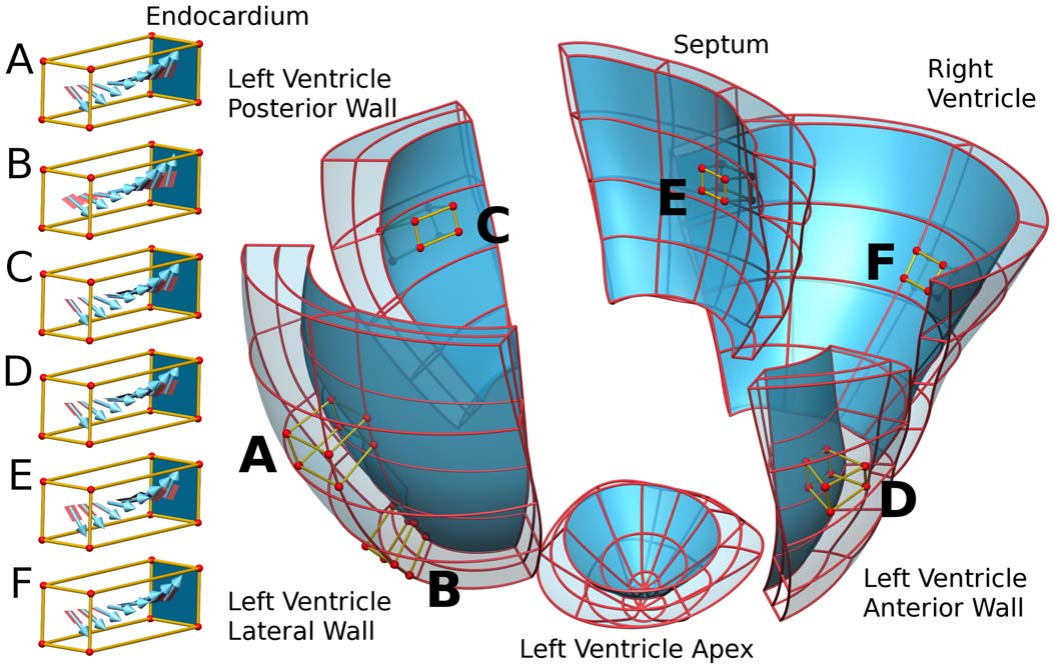
Patient specific model mesh geometry. Cubes show the regional variation in the fibre orientation in the A) mid LV lateral free wall, B) apex LV lateral free wall, C) LV posterior wall, D) LV anterior wall, E) septum and F) RV free wall. The mesh is dissected into labeled regions of the heart.

The model development, equation sets and validation against pressure wire, MRI derived wall motion and endocardial activation times have been described previously [14]. Briefly, end diastolic cine MRI data were manually segmented and fitted to form a tri-cubic Hermite mesh used for simulating mechanical deformations. A second, higher resolution, tetrahedral element mesh, was constructed for simulating cardiac electrophysiology. The fibre orientation was derived from animal studies augmented with human data [14], where available (see Fig. 1). Late enhancement MRI studies were used to identify regions of myocardial scarring. The model heart was separated into two regions: regions with scar and the remaining viable tissue.

Electrical activation was simulated using the mono-domain equations with a human ventricular myocyte cell model [15]. Native activation through the Purkinje system was approximated by stimulating volumes of early activated myocardium, in the LV this was determined by Ensite data and in the RV the Purkinje activation region was determined by projecting the sites of earliest activation from human heart volume activation maps [16] on to the RV wall. Scar tissue was characterised as isotropic low conduction regions. EnSite™ activation data and the QRS duration of the ECG provided the timing and location of stimulation for baseline conditions and were used to fit the conduction parameters. The locations of catheter pacing sites are shown in the supplement (Fig. S2).

Mechanical deformation was simulated using the finite-elasticity equations. The passive material properties of the heart were modelled using a transversely isotropic material law, aligned to the fibre microstructure of the myocardium. Scar was simulated as an isotropic region with increased stiffness. The parameters for the passive material constitutive equation were fitted using the pressure and volume relationship during atria contraction, when the myocardium was assumed to be quiescent.

During isovolumetric contraction (IVC) the volume of the LV and RV cavities were held constant. A Windkessel model provided the pressure-volume relationship for the heart model during ejection, with parameters fitted to the recorded pressure-volume relationship during ejection. Active contraction was simulated through a function of electrical activation time with parameters fitted to the recorded pressure transient.

Simulations were run from 0 ms at the end of diastole to the start of isovolumetric relaxation. A summary of the software and hardware used for model development and simulation is provided in Supplement S1. Because work in the heart is predominantly determined by systole (as opposed to external forces such as venous return and atrial contraction that occur during diastole), the reported results focus on the first half (400 ms) of the cardiac cycle.

### Model Analysis

The model focuses on characterizing the systolic function of a specific patient’s heart to predict the work performed by the heart pre- and post-CRT. The work rate per unit volume was calculated in simulations as the product of the active tension and the SR.

*Work Rate per unit volume (kJm^−3^ms^−1^) = active-tension (kPa) x strain rate (ms^−1^).*

Where the activate tension and strain rate are the second Piola Kirchhoff Stress and the Green strain rate respectively. The work rate can be positive or negative correspond to positive or negative modes of work, as described above. The average work rate in the LV, RV and septum (see Fig. 1) was calculated by integrating the work rate per unit volume over each region and dividing by the size of the region’s volume. This value was integrated over time to give the total work performed by the LV, RV or septum.

The model assumes that minimal energy is lost through viscous dissipation, heat or friction. Therefore the work performed by the heart to eject blood in the RV and LV, calculated from the area enclosed by the respective pressure volume loops, is equal to the sum of the work performed by the cardiac myocytes in the myocardium. The total cardiac myocyte work is equal to the sum of the work performed by the myocardium in the LV, RV and septal regions. This means that the work perfumed to eject blood is directly related to the regional work through.

*RV ejection work + LV ejection work = Myocardium work = LV work + RV work + septum work.*

Changes in the work or work rate in a region of the myocardium therefore result in changes in the work performed to eject blood by the LV or RV.

## Results

**Figure 2:**
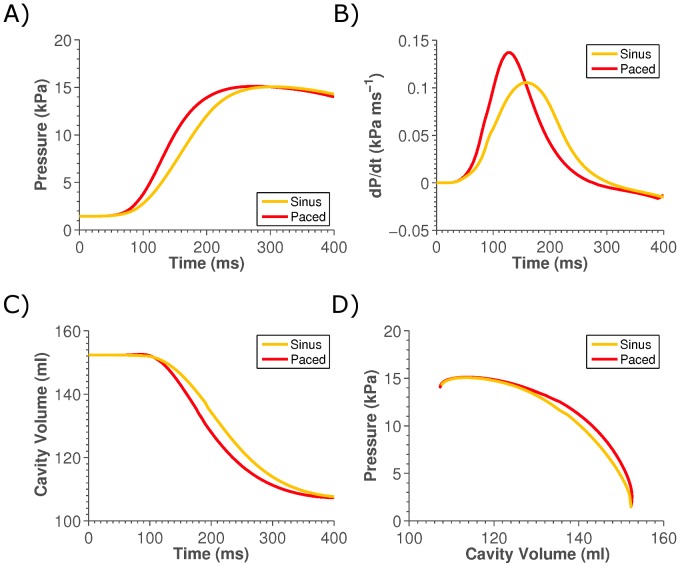
Plot of changes in global metrics of cardiac function following LV pacing. Panel A), B) and D) show the change in left ventricle pressure, rate of pressure development and volume, respectively, on pacing. Panel D) shows the phase plot of pressure and volume, where the area enclosed beneath the lines is equal to the work performed.

Two simulations of systole were performed, corresponding to pre- and post-CRT pacing. The model has been validated previously [14] and further validation of the model is provided in the supplement (Fig. S3 and S4). Fig. 2 shows simulations at base line and the predicted changes following pacing of the pressure, dP/dt, volume and pressure-volume phase plot. Model results demonstrated a 6.54% increase in the work performed by the heart following pacing.

**Figure 3:**
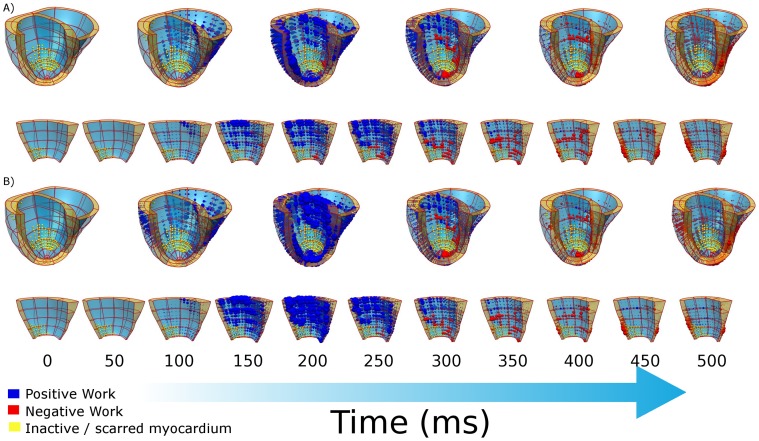
Plot of local work (A) pre- and (B) post-CRT, yellow spheres correspond to regions of scar that perform no work. Blue spheres and red spheres correspond to regions performing positive and negative work, respectively and the size of the sphere corresponds to the magnitude of the work rate. Panels A) and B) show in the first row the local work in the whole heart at 100 ms intervals, and in the second row a close up of the septum at 50 ms intervals.

Fig. 3 shows the regional work performed by the heart during systole pre- and post-pacing. In the LV and septum there is always a fraction of the heart that is performing zero work, which corresponds to the scar region (yellow spheres in Fig. 3). There is a significant increase in the work rate performed by the septum upon CRT, a minor increase in the RV work rate and a slight decrease in the LV work rate. The change in the peak work rate and the total work over systole are summarized in Table 1.

**Table 1:** Peak and total work during systole.

	Region		
Metric	LV	RV	Septum
Work performed during systole by active-tension (kJm^−3^)			
Sinus	−4.93	−3.12	−3.42
Paced	−4.67	−3.39	−4.87
Percentage Change	−5.30	8.62	42.36
Peak average work rate (kJm^−3^ms^−1^)			
Sinus	−0.030	−0.019	−0.019
Paced	−0.028	−0.024	−0.031
Percentage Change	−6.54	21.39	63.88
Peak average SR (ms^−1^)			
Sinus	−0.0010	−0.0012	−0.0010
Paced	−0.0010	−0.0011	−0.0006
Percentage Change	5.49	−6.27	−38.54
Peak average active-tension (kPa)			
Sinus	69.59	61.42	73.55
Paced	70.50	61.39	73.52
Percentage Change	1.30	−0.048	−0.044
Peak negative work volume fraction during IVC			
Sinus	0.094	0.052	0.117
Paced	0.129	0.063	0.201
Percentage Change	37.92	20.91	72.51

**Figure 4:**
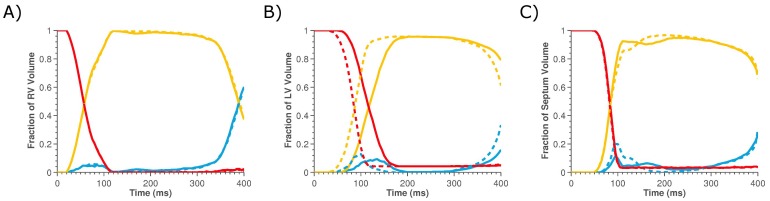
Plots the relative volumes of the A) RV, B) LV and C) septum performing zero work (red lines), positive work that can contribute to pump function (yellow lines) and negative work that opposes pump function (blue lines) for sinus rhythm (solid lines) and LV paced case (dashed lines).

The volumes of the heart that are non-working or are performing positive or negative work are evaluated to determine if the increase in work in the septum and RV regions of the heart following CRT is due to changes in the fraction of the heart in each of these work modes. Fig. 4A–C plots the fraction of the LV, RV or septum performing each mode of work during sinus rhythm and pacing. The changes in the peak volume fraction of the myocardium performing negative work in each region are provided in Table 1.

**Figure 5:**
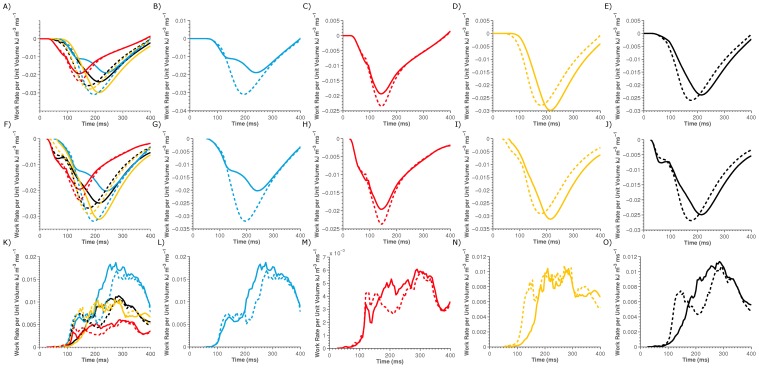
Plot of regional work rates for the LV (yellow lines), RV (red lines), septum (blue lines) and the whole heart (black lines) for sinus rhythm (solid lines) and for LV pacing (dashed lines). Panel A) compares the total work density. Panels B–E) separates Panel A) into regional plots of Septum, RV, LV and whole heart work densities individually for sinus rhythm and LV pacing. Similarly Panel F–J) compares the total work density in volumes of the heart that are performing positive work and Panel L–O) compares the regional work performed in volumes of the heart performing negative work.

To determine if the change in work rates following LV pacing is a result of an increase or a decrease in the magnitude of the peak rate of positive or negative work, respectively, in the LV, RV or septum, each region was separated into volumes performing no work, positive work or negative work. The average work rate in the positive or negative work volumes were calculated and plotted in Fig. 5G–J and 5K–O, respectively. Fig. 5G–O show that the changes in the magnitude of the average work rate in the septum, LV, RV and whole heart following CRT is due to changes in the peak work rate in the volumes performing positive work, as opposed to changes in the work rate in volumes performing negative work.

**Figure 6:**
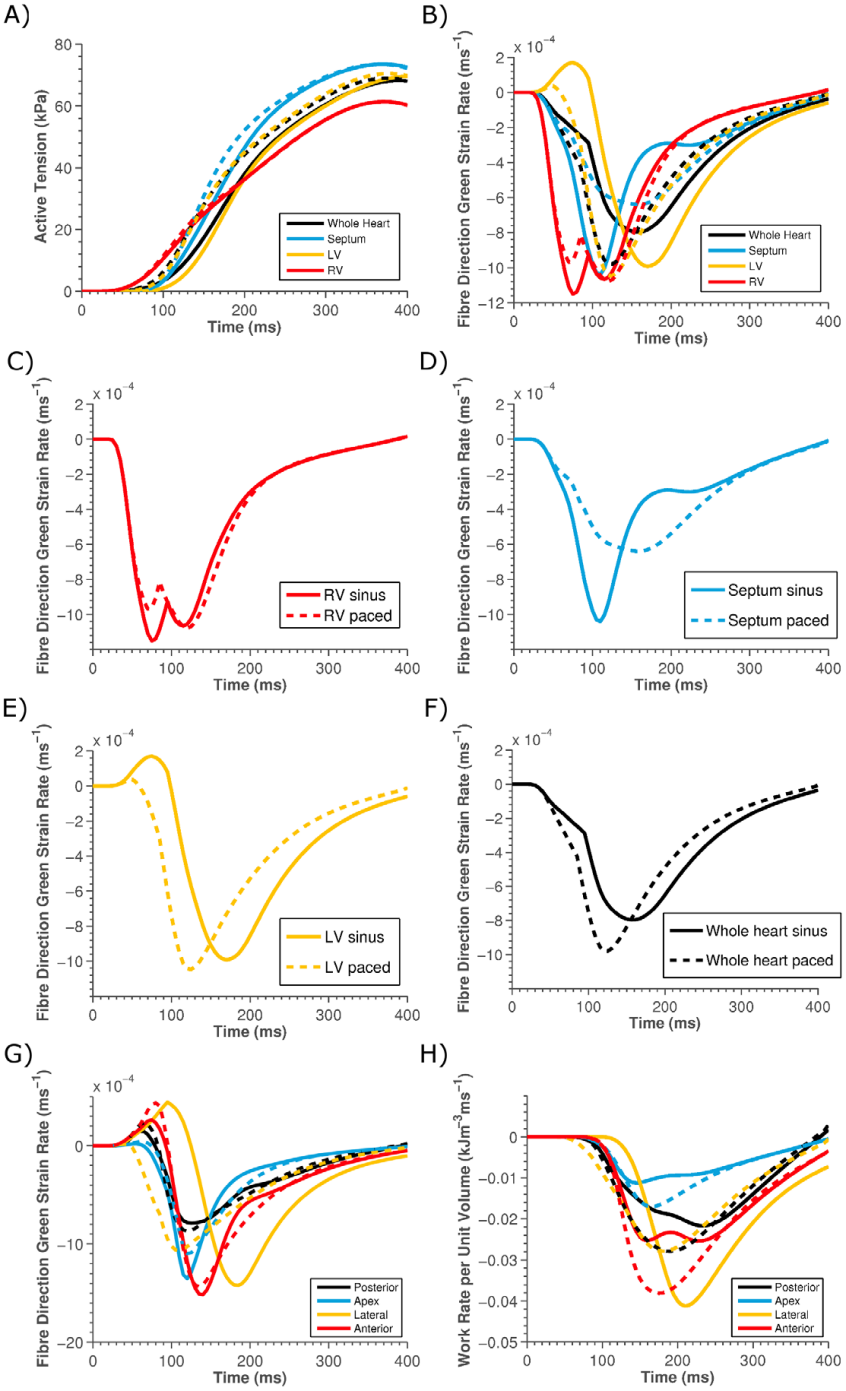
Plot of the average A) active-tension and B) fibre SR in the LV (yellow lines), RV (red lines), septum (blue lines) and the whole heart (black lines) for sinus rhythm (solid lines) and for LV pacing (dashed lines). Panels C–F) show the individual plots from Panel B) for clarity. Panels G) and H) show the average work rate and the average SR, respectively, in the posterior (black), apex (blue), lateral (yellow) and anterior (red) LV free wall for sinus (solid) and paced (dashed) stimulation.

To determine if either SR or active-tension (the product of which determines work) was responsible for the change in work rate the average SR and active-tension was calculated for the septum, RV, LV and the entire heart. These results are summarized in Table 1 and Fig. 6 and show that the average active-tension in each region remains relatively constant (Fig. 6A), in comparison to the significant change in average SR (Fig. 6B).

**Figure 7:**
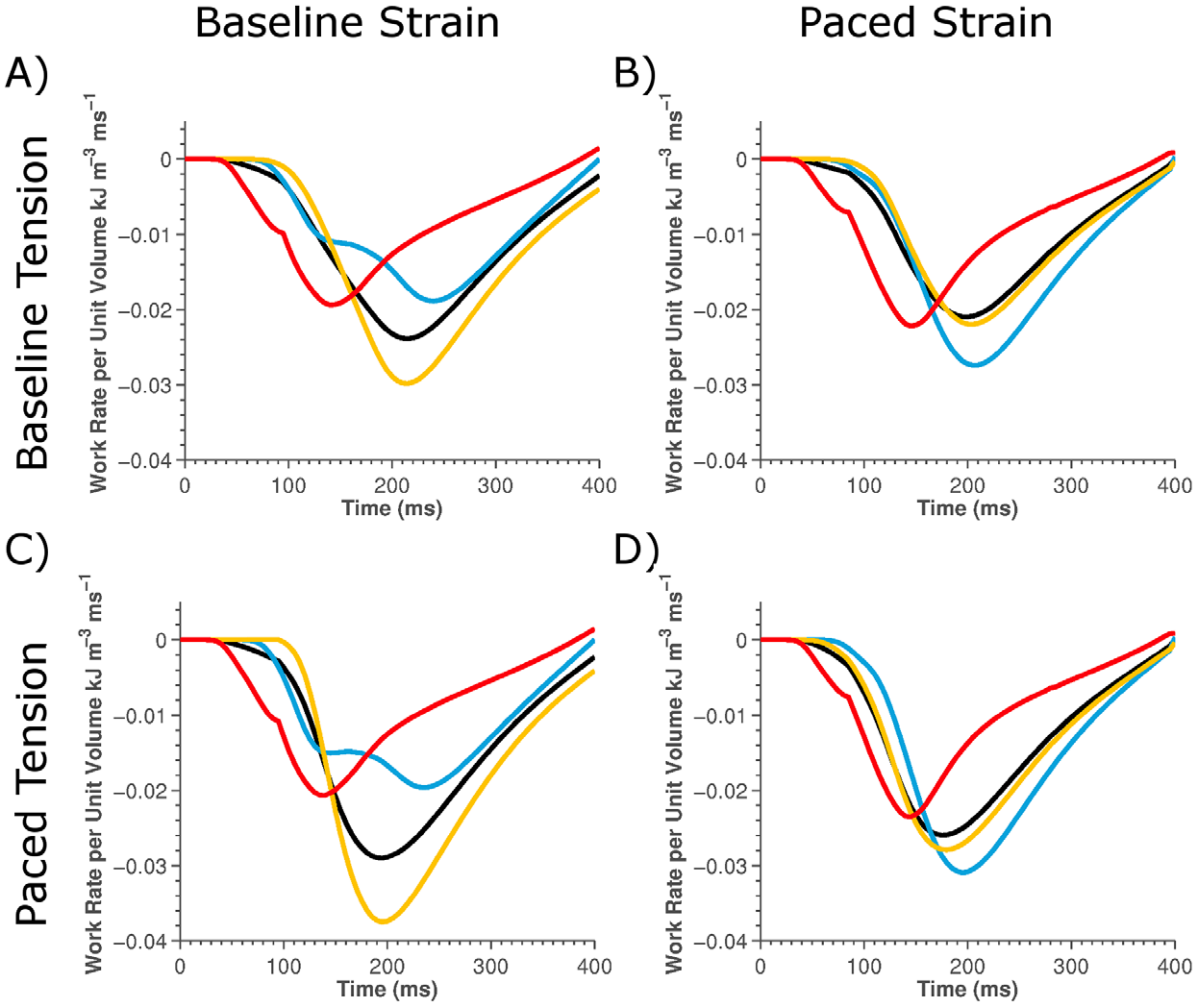
Plot off the regional work rate calculated in the LV (yellow lines), RV (red lines), septum (blue lines) and the whole heart (black lines). The regional work rate was calculated using the SR from the sinus and paced model for the left and right plots, respectively and the tension from the sinus and paced models for the top and bottom plots, respectively.

To confirm that the change in strain patterns as opposed to changes in tension following pacing cause the changes in the LV, RV and septum work rate we evaluated the work rate in the baseline model but using either the SR or active-tension values from the paced model (Fig. 7). This allows us to evaluate the impact of only changing SR or active-tension on the regional work rates.

Following pacing the LV and septum have similar work rates (Fig. 5G and 5I) and perform similar average work (Table 1), this belies the fact that they have very different average regional SRs (Fig. 6D–E). The spatial variation in the work and SRs across the LV were evaluated by calculating the work and SRs in the anterior, posterior, lateral and apical regions (Fig. 6G–H).

## Discussion

The model we have developed links deformation, pressure and electrical activation data with integrative calculations of regional cardiac work. Through this method we are able to investigate the underlying changes in cardiac function that produce changes in regional work observed following CRT.

The model shows a significant (30.4%) increase in the maximum dP/dt in the LV, compared with the 40% increase recorded. The model predicted a nominal increase in peak systolic pressure of 0.3% compared with a small increase of 4.1% recorded for the patient. Although the model underestimates both these measurements, this is likely to be a result of the change in cardiac frequency from 1.15 Hz sinus rhythm to 1.67 Hz when the heart is being paced in the clinic, as the model does not simulate the frequency dependence of the heart. There is only a nominal change in the ejection fraction following pacing in the model, consistent with experimental observations [17] but there is a significant variation in regional deformation and work as seen previously. The model shows a net increase in systolic work over the whole heart of 6.54%. This change is less than previous measurements of ∼13% [1], this difference may be attributable to the previous measurements being recorded 58±26 weeks following CRT or that the average QRS duration for the previous study was 172 ms as opposed to 154 ms for the current patient and CRT outcome is weakly correlated with QRS duration [18].

In the whole heart model the peak positive work rate increased by 8.7%. This was a result of a significant regional increase in the peak work rate in the septum (63.9%) and RV (21.4%) compared with a minor decrease in the LV rate (−6.5%), resulting in the work rates of the septum and LV being more homogeneous. Assuming a correlation between fibre stress–strain work loops and oxygen consumption [9], [19], these changes in regional peak work rate and work during systole predicted by the model (see Table 1) are qualitatively consistent with experimental observations of changes in regional oxidative metabolism following CRT, from previous studies [1], [2], [3], [4].

Fig. 4 shows that pacing increases the peak volume fraction of the heart performing negative work in both the LV and septum from 9.4% to 12.9% and 11.7% to 20.0%, respectively, suggesting that CRT does not improve cardiac efficiency by reducing the volume of the myocardium that is performing negative work. The fraction of the RV performing each type of work is insensitive to pacing; this is attributed to the absence of any change in activation times in the RV between the pre and post CRT models. This significant increase in the fraction of the septum performing negative work is due to the increased stretching of early activated septal fibers in the presence of the contracting LV, which is invariably not present during sinus rhythm due to the presence of LBBB. The changes in the work rates in the LV, RV and septum in the model is thus mainly attributed to changes in the average magnitude of the work rate in the volumes performing positive work. These results combined with the regional changes in peak work rate with pacing, suggests that CRT enables viable, working myocardium to work more effectively as opposed to reducing the deleterious effects of regions performing negative work.

The results showed that there was nominal (<2%) change in the average peak active-tension in any region, but that there was a significant change in the SR following pacing (see Fig. 6). Fig. 7 shows that the rate of work in the septum only significantly increased when work was calculated using the SR from the paced model, independent of tension being calculated from the sinus or paced model, confirming that it is changes in SR and not active-tension with pacing that causes the significant change in septum work rate.

Surprisingly, looking at a regional analysis, the septum had a significant decrease in the peak SR despite a significant increase in the peak rate of work upon pacing (Fig. 6D). Similarly, despite a minor decrease in the peak work rate in the LV upon pacing, there was as slight increase in the maximum SR (Fig. 6E). Hence following CRT, despite the LV and the septum having a comparable peak work rate and performing comparable amounts of work per volume, the mechanism by which this is achieved appears different in each region. In the LV there is a higher average SR at a lower average stress and in the septum there is a slower average SR but this occurs during a period of higher active-tension.

Analyzing the work and strain in each region of the LV shows that the anterior and posterior regions of the LV free wall had strain and work rate transients consistent with the septum transients (see Fig. 6G–H). As previously observed experimentally [11], we see that it is only the LV lateral wall, the region local to the pacing electrode, that has a significant decrease in work rate following pacing. This reduction in work close to the pacing electrode can be attributed to local regions contracting rapidly at relatively low active-tension, thus limiting the work rate.

These results suggest that it is not the magnitude of the rate of shortening but the timing relative to the level of active-tension that is important in this patient for the heart to generate significant levels of work. In the case of the septum during sinus rhythm, the period of early rapid shortening coincides with a phase of low active-tension. When the heart is paced the contraction of the septum is synchronized with the contraction in the rest of the LV. The LV provides the septum with resistance to contract against, slowing the average SR in the septum and as a result the septum continues to contract while generating significant amounts of active-tension to produce an increase in work rate.

### Limitations

The intrinsic complexity of the model necessitates a significant quantity of high quality imaging and diagnostic data recorded from multiple modalities. To ensure that this data is internally consistent we have fitted the model to a single patient data set. The patient’s attributes are representative of the CRT population; however, this does not ensure that the model analysis reflects the average CRT heart. Although the results from this study must be interpreted in this context, by only using data from a single patient we are able to remove any confounding effects of fitting the model to potentially incompatible values taken from population averages. To show that the patient specific parameters did not affect the study conclusions a sensitivity analysis was performed and is presented in the online supplement (Fig. S1 and Table S1).

The results presented here are data driven and hence the model and analysis only consider the acute response to pacing, where data was available to parameterize and validate the model. It is important to note that acute changes may not predict reverse remodeling in CRT patients. Recent studies have of the predictive qualities of the acute hemodynamic response, as quantified by percentage change in maximum LV dP/dt, have reported conflicting results, with Bogaard et al., [20] refuting and Duckett et al., [21] confirming a link between long term response and an acute change in maximum LV dP/dt. Hence, the results from this study, although relevant for the acute response, should be extrapolated to long term CRT outcomes with caution.

The model was able to replicate changes in regional work distributions but did not predict an increase in whole heart efficiency following CRT, as observed clinically [1], [10], [22], although, this increased efficiency may be dependent on the type of HF [3]. In the model the whole heart efficiency remains constant following CRT, which could be the result of a mechanism not captured by the model. Alternately the redistribution of work and the acute improvement in efficiency following CRT could both be the result of the redistribution of stress and strain, however, assumptions embedded in the model have obfuscated this link. Both these scenarios are discussed in detail in Supplement S1.

What the model does demonstrate is that it is possible that the change in whole heart efficiency and the redistribution of energy consumption following CRT do not have to be explained by a common mechanism. This is consistent with observations of no improvement in whole heart efficiency in the presence of a trend towards regional work redistribution in ischemic dilated cardiomyopathy patients following CRT [3].

### Conclusions

In a patient specific model of CRT, improved septal work rate following CRT is the result of resistance to septal contraction provided by the LV free wall. This results in slower septal shortening over a longer period, allowing the septum to contract while generating higher levels of active-tension producing a higher work rate.

## Supporting Information

Figure S1 Plot of percentage change in peak work rate in the A) whole heart, B) LV, C) RV and D) septum. Factors with light and dark bars correspond to −10% (light) and +10% (dark) changes in a factor. Factors with a single bar correspond to binary changes. Factor labels are provided in Table S1.
(PNG)

Figure S2 Catheter stimulation sites in the model. The RV site location is indicated but it is located on the apex of the RV septum wall. The cream geometry indicates the endocardium geometry extracted from Ensite.
(PNG)

Figure S3 Panel A), B), C) and D) show compare the Ensite endocardial activation maps (top of panel) with simulated endocardial activation (bottom of panel) and base line (red line) and paced (yellow line) pressure catheter measurements with simulations for right ventricle pacing, left ventricle endocardium pacing, left ventricle coronary sinus, left ventricle endocardium and right ventricle pacing, and left ventricle endocardium and right ventricle pacing. In panel A) the LV free wall is labelled with a 1 and the septum labelled with a 2.
(PNG)

Figure S4 Heart geometry showing the segments used to evaluate local longitudinal velocity. The blue segment corresponds to the LV free wall epicardium, green to the mid LV free wall, gold to the LV free wall endocardium, magenta to the LV septum and cyan to the RV septum.
(JPG)

Table S1 List of factors evaluated in sensitivity analysis and corresponding index for factors in Fig. S1.
(DOC)

Supplement S1 Online Supplement.
(DOCX)
