# Supplementary figures and images for: Analyses of the Redistribution of Work following Cardiac Resynchronisation Therapy in a Patient Specific Model
Source: PLoS One. 2012 Aug 28;7(8):e43504. doi: 10.1371/journal.pone.0043504 (PMC3429501; doi:10.1371/journal.pone.0043504)

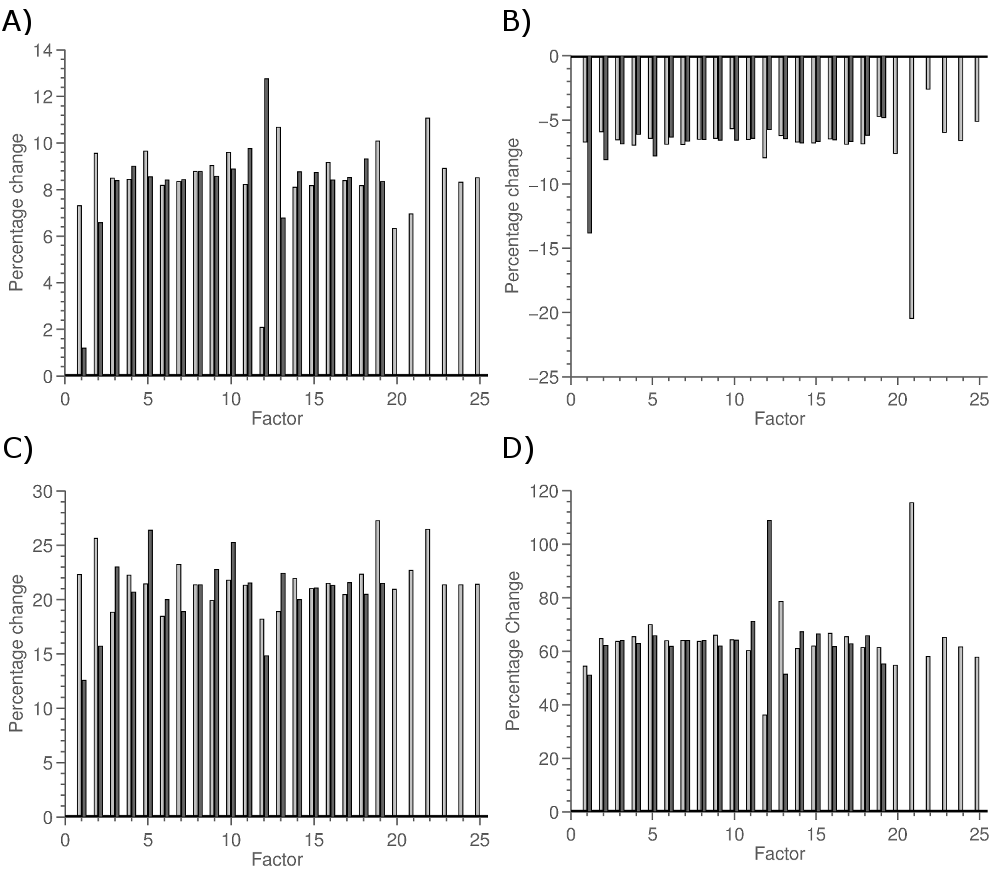

Supplement: Figure S1 — Plot of percentage change in peak work rate in the A) whole heart, B) LV, C) RV and D) septum. Factors with light and dark bars correspond to −10% (light) and +10% (dark) changes in a factor. Factors with a single bar correspond to binary changes. Factor labels are provided in Table S1. (PNG) [file pone.0043504.s001.png]

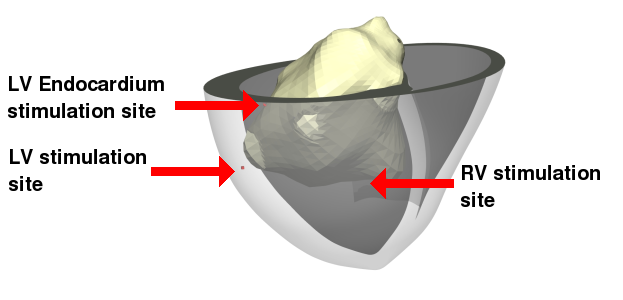

Supplement: Figure S2 — Catheter stimulation sites in the model. The RV site location is indicated but it is located on the apex of the RV septum wall. The cream geometry indicates the endocardium geometry extracted from Ensite. (PNG) [file pone.0043504.s002.png]

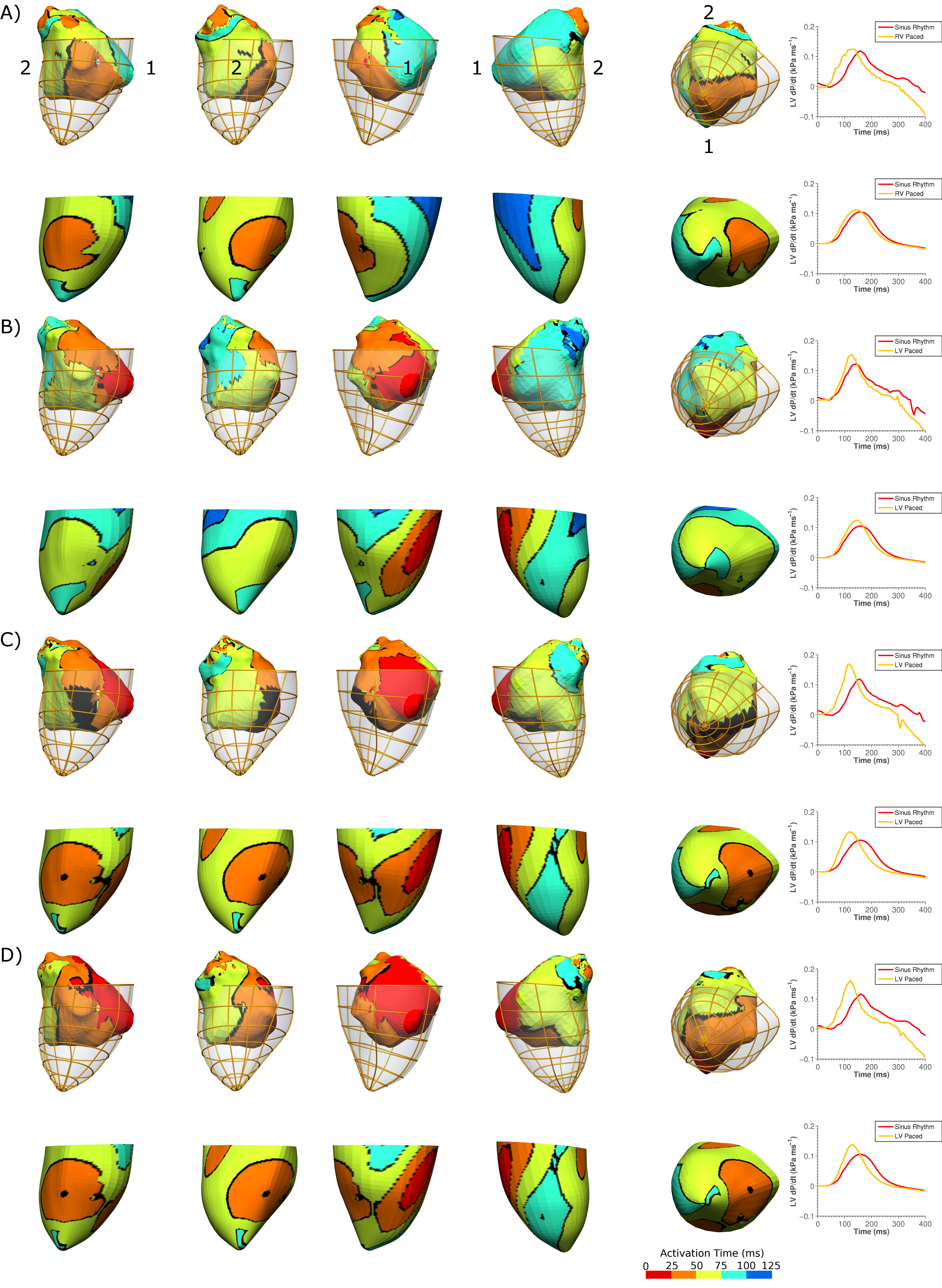

Supplement: Figure S3 — Panel A), B), C) and D) show compare the Ensite endocardial activation maps (top of panel) with simulated endocardial activation (bottom of panel) and base line (red line) and paced (yellow line) pressure catheter measurements with simulations for right ventricle pacing, left ventricle endocardium pacing, left ventricle coronary sinus, left ventricle endocardium and right ventricle pacing, and left ventricle endocardium and right ventricle pacing. In panel A) the LV free wall is labelled with a 1 and the septum labelled with a 2. (PNG) [file pone.0043504.s003.png]

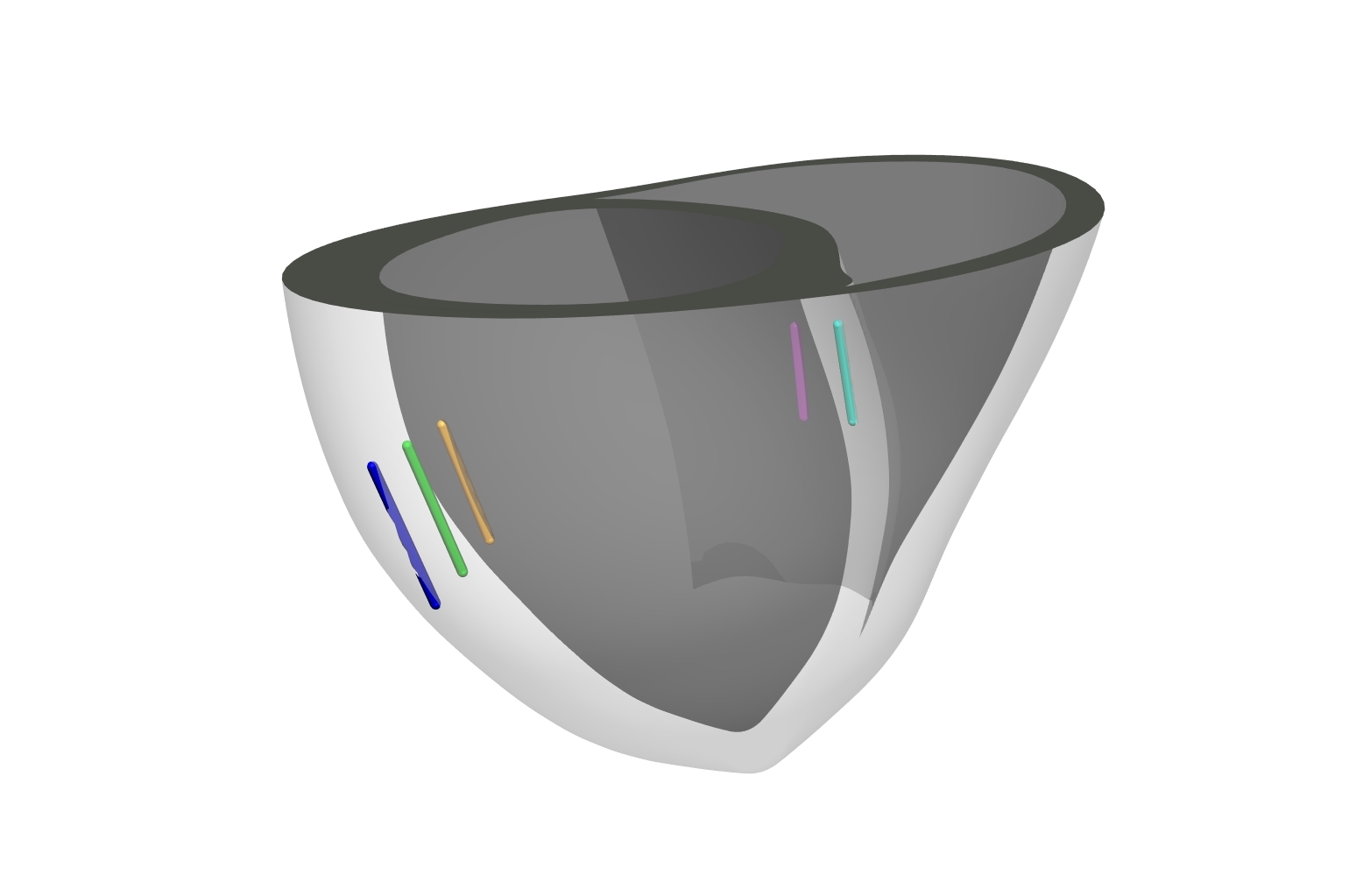

Supplement: Figure S4 — Heart geometry showing the segments used to evaluate local longitudinal velocity. The blue segment corresponds to the LV free wall epicardium, green to the mid LV free wall, gold to the LV free wall endocardium, magenta to the LV septum and cyan to the RV septum. (JPG) [file pone.0043504.s004.jpg]
