# Supplementary material for: Analyses of the Redistribution of Work following Cardiac Resynchronisation Therapy in a Patient Specific Model
Source: PLoS One. 2012 Aug 28;7(8):e43504. doi: 10.1371/journal.pone.0043504 (PMC3429501; doi:10.1371/journal.pone.0043504)
Supplement: Table S1 — List of factors evaluated in sensitivity analysis and corresponding index for factors in Fig. S1. (DOC) [file pone.0043504.s005.doc]

| Factor | Index |
| --- | --- |
| Fibre angle | 1 |
| Aortic pressure | 2 |
| Mechanics stiffness (C_o_ Guccione law) | 3 |
| Compliance in Windkessel model | 4 |
| Conduction in the fibre direction | 5 |
| Conduction in the transverse fibre direction | 6 |
| End diastolic volume | 7 |
| Systolic resistance in Windkessel model | 8 |
| Aortic resistance in Windkessel model | 9 |
| Nonlinear stiffness (α Guccione law) | 10 |
| Duration of active tension transient | 11 |
| Stretch at which active tension reaches zero | 12 |
| Level of length-dependent tension | 13 |
| Scalar for length-dependent rate of tension development | 14 |
| Base active tension development rate | 15 |
| Rate of relaxation | 16 |
| Peak tension | 17 |
| Septum active tension | 18 |
| Septum stiffness | 19 |
| Bi-domain model of electrophysiology | 20 |
| Right branch bundle block | 21 |
| No scar region | 22 |
| No change in active tension model in scar region | 23 |
| No change in electrophysiology model in scar region | 24 |
| No change in passive mechanics model in scar region | 25 |

Table S1: List of factors evaluated in sensitivity analysis and corresponding index for factors in Fig. S1.
