# Supplementary material for: Analyses of the Redistribution of Work following Cardiac Resynchronisation Therapy in a Patient Specific Model
Source: PLoS One. 2012 Aug 28;7(8):e43504. doi: 10.1371/journal.pone.0043504 (PMC3429501; doi:10.1371/journal.pone.0043504)
Supplement: Supplement S1 — Online Supplement. (DOCX) [file pone.0043504.s006.docx]

**Supplement**

**Patient Characteristics**

The patient included in this study is representative of a significant portion of patients partaking in large clinical studies. The patients QRS is 154ms compared with 150-180ms [[1](#_ENREF_1),[2](#_ENREF_2),[3](#_ENREF_3)], resting heart rate is 68 beats per minute compared to 71±12.4 [[3](#_ENREF_3)], left ventricular ejection fraction is 25% compared with 20-29% [[1](#_ENREF_1),[2](#_ENREF_2),[3](#_ENREF_3)], six minute walk test is 206m compared to 243±129m [[3](#_ENREF_3)] and 274m [[2](#_ENREF_2)], the patient NYHA class was III and the patient had an ischemic myopathy in common with 40-64% of patients in the large CRT clinical trials [[1](#_ENREF_1),[2](#_ENREF_2),[3](#_ENREF_3)]. The site of earliest and last activation, derived from Ensite maps, were in the basal septum and posterior lateral free wall consistent with previous studies [[4](#_ENREF_4)].

**Sensitivity**

The current model represents an approximation of a patient’s heart. The model is intrinsically a simplified representation hence there is inevitably error in the model predictions. The model personalization process, the clinical data itself and inter patient variability all have the capacity to introduce further error into the model. Although this error is unavoidable it is possible to determine the sensitivity of the study conclusions to model assumptions, parameters and disease state. We have performed a sensitivity analysis by varying model parameters that represent the model passive and active mechanical properties, the after-load and pre-load models, the fibre orientation and the electrical conduction by ±10% and calculating how changes in these parameters alter the percentage change in peak work rate in the whole heart and in the RV, septum and LV regions with pacing. We have also evaluated the sensitivity of the model to the presence of scar, as well as how the scar is represented by the passive mechanics, active mechanics and electrical conduction. To test if the baseline activation pattern affected results we have also simulated a left and right bundle block baseline case. The current model of electrical activation used the simplified mono-domain equations, to test if this simplification affected study conclusions simulations with the bi-domain model were also performed. It is also possible that spatial variations affect model results. Spatial variations in fibrosis, perfusion or adrenergic innervations could be present in the patient’s heart and affect simulation results. To investigate the effect of heterogeneities on the model we consider the case where the septum properties differ from the rest of the ventricles. To estimate the potential effects of regional fibrosis in the septum, simulations with increased septum stiffness were preformed. The potential effects of reduced perfusion in the septum were approximated by decreasing stiffness in the septum, although this ignores the temporal effects of perfusion it provides a first order approximation of the expected effects of decreased perfusion on electromechanics. The potential impact of increased or decreased adrenergic innervations in the septum is approximated by increased or decreased active tension in the septum. The sensitivity results are summarised in Figure S1, the factors are labelled in Table S1.

Perturbing model parameters, boundary conditions and assumptions invariably alters model results, but as the results show here despite a significant perturbation in a wide range of factors the model always has the same qualitative results. The peak work rate increases in the whole heart (Fig. S1A), decreases in the LV (Fig. S1B), increases in the RV (Fig. S1C) and increases in the septum (Fig. S1D), hence although the magnitude of the change is altered with different parameter combinations the direction of the change is consistent across all parameter sets tested.

**Validation**

To further validate the model we compared simulated and clinically measured results of the electrical and mechanics response of the heart to different pacing locations and combinations. Figure S3 shows the changes in activation patterns and pressure transients for pacing from the right ventricle only (Fig. S3A), the left ventricle endocardium only (Fig. S3B), the left ventricle endocardial, right ventricle and left ventricle coronary sinus pacing sites (Fig. S3C) and the left ventricle endocardium in conjunction with the right ventricle (Fig. S3D). The location sites in the model and there locations with respect to the Ensite mapping array LV endocardium geometry are shown in Fig. S2.

Simulations of pacing from the endocardium only, as shown in Figure S3B, did not result in fusion with intrinsic septal activation. For this reason, in these simulations the septum activation was set to zero. The biventricular pacing combination with stimulation of the right ventricle and left ventricle coronary sinus did not result in capture at the left ventricle pacing site as evident by the late activation in the lateral wall observed in the Ensite map and so was not included in the model validation. In Figure S3C and D the activation patterns appear almost identical in the first two panels. For these two protocols the only difference in the pacing protocol is absence of LV coronary sinus pacing in D. For this reason the activation patterns in the region of the septum and RV are similar.

Using the available patient data set it is possible to validate the models of electrical activation and deformation for baseline activation, as well as validate the model of electrical activation and global hemodynamics for multiple pacing cases. However, no information is available for this specific patient characterising cardiac deformation post CRT. To confirm that the patient deformation patterns are consistent with previous clinical trials we compare measurements of deformation and timing in the patient model with literature values.

Studies have reported changes in peak systolic velocities in the LV lateral free wall and the septum pre and post CRT using tissue Doppler imaging. Evaluating these measurements directly in the model is nontrivial; the definition of longitudinal motion is dependent on the orientation and location of the echo probe with regards to the patient’s heart, also the boundary conditions in the model restrict rigid body motion but do not constrain the heart to move in a coordinate system with respect to the rest of the body, which is not simulated. Also it is not possible to uniquely identify the region of interest used to evaluate the longitudinal velocities in the original study in the current model, this could make comparison particularly challenging in the presence of large velocity gradients. To provide a comparison between the model results and these measurements we have taken the lateral velocity recorded in echocardiograms as a measure of the rate of lateral shortening of the myocardium. We have therefore evaluated the shortening velocity of linear longitudinal segments between nodes in the mechanics mesh across the heart wall at the lateral free wall and the septum, as indicated in Fig. S4, to provide a metric for qualitative comparison with Doppler measurements.

The model predicts peak segment shortening velocities of -1.43, -0.65 and -0.74 cms^-1^ in the endocardium, mid and epicardium lateral free wall, respectively and -0.13 and -0.07 cms^-1^ in the LV and RV septum, respectively, during baseline rhythm. During pacing these values change to -0.64, -0.50, -1.21 cms^-1^ in the endocardium, mid and epicardium lateral free wall, respectively and -0.34 and -0.31 cms^-1^ in the LV and RV septum, respectively. The model predicts that the peak segment shortening velocities decrease in the endocardial and mid lateral free wall, while increasing in the lateral free wall epicardium. This is consistent with changes in lateral peak velocities reported by Breithardt et al., [[5](#_ENREF_5)], who observed peak velocities decreasing in the mid LV lateral free wall and an increasing in the septum with pacing. Other observations by Bax et al., [[6](#_ENREF_6)] report increases in peak lateral velocity in both the septum and LV lateral free wall upon pacing. The model replicates the increase in septum peak lateral velocity, but cannot readily reconcile the results of Bax et al, [[6](#_ENREF_6)] and Breithardt et al., [[5](#_ENREF_5)]. We can also compare the duration of IVC predicted by the model with clinical measurements. The model predicts a decrease in IVC time from 95 to 80ms acutely after CRT compared to changes of 121±29ms to 99±24ms observer clinically after 1 week of pacing [[7](#_ENREF_7)] and IVC times of 122±57 to 93±42ms and 138±57 to 100±37ms [[8](#_ENREF_8)], in non responders and responders, respectively, following three months of pacing. Although the model predictions do not quantitatively match the average measurements the change in IVC is in the correct direction and the model values do fall within the error bars of both studies.

Results in previous studies [[9](#_ENREF_9)] and this supplement demonstrate that the proposed model is capable of replicating electrical and mechanical direct measurements and is consistent with literature results both pre and post CRT. Although this does not fully validate the model, taken in tandem with the comprehensive sensitivity analysis it provides confidence that the study results are robust with respect to the model assumptions and personalization.

**Whole Heart Efficiency Extended Discussion**

The model was able to replicate changes in regional work distributions but did not predict an increase in whole heart efficiency following CRT, as observed clinically [[10](#_ENREF_10),[11](#_ENREF_11),[12](#_ENREF_12)], however, this increased efficiency may be dependent on the type of HF [[13](#_ENREF_13)]. For the efficiency of the current model to improve there would need to be a decrease in the amount negative work performed following CRT. However, this was not the case, despite observing a 6.54% increase in work during systole following CRT the amount of work spent on stretching tension generating myocardium remained constant at approximately 2% of the total work. This means that the whole heart efficiency remains constant in the model following CRT, which could be attributed to one of two possibilities. The first possibility is that the redistribution of work and the acute improvement in efficiency following CRT are the result of two independent changes in cardiac function. The model predicts that the redistribution of work is due to changes in the spatial and temporal redistribution of stress and strain, whereas the acute change in whole heart efficiency could be the result of a mechanism not captured by the model.

Alternately the redistribution of work and the acute improvement in efficiency following CRT could both be the result of the redistribution of stress and strain, however, assumptions embedded in the model have obfuscated this link. In the model analysis we have assumed a simple correlation between oxygen consumption and work. However, work is likely to have a complex relationship with the consumption of oxygen and this relationship is likely to be altered by the metabolic changes present in HF[[14](#_ENREF_14)]. Specifically the model does not assume a higher energy cost per unit of work for generating tension at higher stress or for rapid shortening, as occurs in the lateral free wall and in the septum, respectively, pre-CRT. Both of these mechanisms have been proposed to explain the improved global efficiency in the heart following CRT [[11](#_ENREF_11),[15](#_ENREF_15)]. Furthermore, the model of contraction assumes that no active-tension is generated during diastole, which may not be the case in HF where elevated diastolic cytosolic calcium [[16](#_ENREF_16)] will increase diastolic tone with a concurrent increase in diastolic oxygen consumption [[17](#_ENREF_17)]. Rapid movements in the heart may result in energy loss through viscous dissipation in either the blood in the LV cavity [[18](#_ENREF_18),[19](#_ENREF_19)] or the myocardium [[20](#_ENREF_20)]. None of these potential consumers of energy was accounted for in this study, as they could not be readily characterized by the already extensive clinical data set used to personalize the model. The absence of these mechanisms may explain the difference between the lack of improved whole heart efficiency in the model compared with clinical studies.

**References**

1. Cleland JGF, Daubert JC, Erdmann E (2005) The Effect of Cardiac Resynchronization on Morbidity and Mortality in Heart Failure. ACC Current Journal Review 14: 20-20.

2. Bristow MR, Saxon LA, Boehmer J, Krueger S, Kass DA, et al. (2004) Cardiac-Resynchronization Therapy with or without an Implantable Defibrillator in Advanced Chronic Heart Failure. NEJM 350: 2140-2150.

3. Young JB, Abraham WT, Smith AL, Leon AR, Lieberman R, et al. (2003) Combined Cardiac Resynchronization and Implantable Cardioversion Defibrillation in Advanced Chronic Heart Failure: The MIRACLE ICD Trial. JAMA 289: 2685-2694.

4. Tanaka H, Hara H, Adelstein EC, Schwartzman D, Saba S, et al. Comparative Mechanical Activation Mapping of RV Pacing to LBBB by 2D and 3D Speckle Tracking and Association With Response to Resynchronization Therapy. J Am Coll Cardiol Img 3: 461-471.

5. Breithardt O-A, Stellbrink C, Herbots L, Claus P, Sinha AM, et al. (2003) Cardiac resynchronization therapy can reverse abnormal myocardial strain distribution in patients with heart failure and left bundle branch block. Journal of the American College of Cardiology 42: 486-494.

6. Bax JJ, Molhoek SG, van Erven L, Voogd PJ, Somer S, et al. (2003) Usefulness of myocardial tissue Doppler echocardiography to evaluate left ventricular dyssynchrony before and after biventricular pacing in patients with idiopathic dilated cardiomyopathy. The American Journal of Cardiology 91: 94-97.

7. Yu C-M, Chau E, Sanderson JE, Fan K, Tang M-O, et al. (2002) Tissue Doppler Echocardiographic Evidence of Reverse Remodeling and Improved Synchronicity by Simultaneously Delaying Regional Contraction After Biventricular Pacing Therapy in Heart Failure. Circulation 105: 438-445.

8. Yu C-M, Fung W-H, Lin H, Zhang Q, Sanderson JE, et al. (2003) Predictors of left ventricular reverse remodeling after cardiac resynchronization therapy for heart failure secondary to idiopathic dilated or ischemic cardiomyopathy. The American Journal of Cardiology 91: 684-688.

9. Niederer SA, Gernot P, Chinchapatnam P, Ginks M, Lamata P, et al. (2010) Length-Dependent Tension in the Failing Heart and the Efficacy of Cardiac Resynchronisation Therapy. Cardiovascular Research 10.1093/cvr/cvq318.

10. Ukkonon H, Beanlands RSB, Burwash IG, de Kemp RA, Nahmias C, et al. (2003) Effect of Cardiac Resynchronization on Myocardial Efficiency and Regional Oxidative Metabolisim. Circulation 107: 28-31.

11. Nelson GS, Berger RD, Fetics BJ, Talbot M, Spinelli JC, et al. (2000) Left Ventricular or Biventricular Pacing Improves Cardiac Function at Diminished Energy Cost in Patients With Dilated Cardiomyopathy and Left Bundle-Branch Block. Circulation 102: 3053-3059.

12. Sundell J, Engblom E, Koistinen J, Ylitalo A, Naum A, et al. (2004) The effects of cardiac resynchronization therapy on left ventricular function, myocardial energetics, and metabolic reserve in patients with dilated cardiomyopathy and heart failure. Journal of the American College of Cardiology 43: 1027-1033.

13. Lindner O, Vogt Jr, Kammeier A, Wielepp P, Holzinger J, et al. (2005) Effect of cardiac resynchronization therapy on global and regional oxygen consumption and myocardial blood flow in patients with non-ischaemic and ischaemic cardiomyopathy. European Heart Journal 26: 70-76.

14. Ingwall JS, Weiss RG (2004) Is the Failing Heart Energy Starved?: On Using Chemical Energy to Support Cardiac Function. Circ Res 95: 135-145.

15. Kawaguchi M, Murabayashi T, Fetics BJ, Nelson GS, Samejima H, et al. (2002) Quantitation of basal dyssynchrony and acute resynchronization from left or biventricular pacing by novel echo-contrast variability imaging. Journal of the American College of Cardiology 39: 2052-2058.

16. Gwathmey JK, Copelas L, MacKinnon R, Schoen FJ, Feldman MD, et al. (1987) Abnormal intracellular calcium handling in myocardium from patients with end-stage heart failure. Circ Res 61: 70-76.

17. Baller D, Wolpers HG, Hoeft A, Korb H, Rösick A, et al. (1984) Increase of myocardial oxygen consumption due to active diastolic wall tension. Basic Research in Cardiology 79: 176-185.

18. Pedrizzetti G, Domenichini F (2005) Nature Optimizes the Swirling Flow in the Human Left Ventricle. Physical Review Letters 95: 108101.

19. Steendijk P, Tulner SA, Bax JJ, Oemrawsingh PV, Bleeker GB, et al. (2006) Hemodynamic Effects of Long-Term Cardiac Resynchronization Therapy: Analysis by Pressure-Volume Loops. Circulation 113: 1295-1304.

20. Noble MIM, Milne ENC, Goerke RJ, Carlsson E, Domenech RJ, et al. (1969) Left Ventricular Filling and Diastolic Pressure-Volume Relations in the Conscious Dog. Circ Res 24: 269-283.
